# Cytogenetic significance of chromosome 17 aberrations and P53 gene mutations as prognostic markers in oral squamous cell carcinoma

**DOI:** 10.1186/s13000-015-0232-1

**Published:** 2015-02-22

**Authors:** Walid Zedan, Mohamed I Mourad, Sherin M Abd El-Aziz, Nagla M Salamaa, Asem A Shalaby

**Affiliations:** Department of Oral and maxillofacial Pathology, Faculty of Dentistry, Mansoura University, Mansoura, Egypt; Department of Clinical Pathology-Hematology, Faculty of Medicine, Mansoura University, Mansoura, Egypt; Department of General Pathology, Faculty of Medicine, Mansoura University, Mansoura, Egypt

**Keywords:** Oral squamous cell carcinoma, P53 gene, P53 immunoreactivity, Chromosome 17, FISH technique

## Abstract

**Background:**

Cytogenetic analysis has detected an accumulation of genetic lesions in oral cancers. Numerical changes in chromosome 17 might be associated with an up-regulation of p53 gene, and could contribute to critical events in carcinogenesis. The aim of this study was to reveal possible correlations between the numerical aberrations of chromosome 17, deletion or amplification of the P53 gene and histological grading in patients with oral squamous cell carcinoma (OSCC).

**Methods:**

This study was performed retrospectively on anonymous forty paraffin embedded specimens diagnosed with a primary OSCC. Sections were prepared for p53 immunohistochemical staining and FISH technique evaluation.

**Results:**

All studied cases showed a positive nuclear staining with different indices for the p53 protein. Furthermore, statistical analysis showed a significant difference between all histological types of OSCC. In term of P53 immunoreactivity well differentiated OSCC showed the highest, whereas poorly differentiated showed weakest. Regarding chromosome 17 aberrations and p53 gene mutations, Spearman correlation test revealed a statistical significant positive correlation between chromosome 17 abnormalities and p53 gene mutations as well as with the immunohistochemical expression of p53 proteins. Moreover, the positive association between p53 gene mutations and the expression of p53 protein was statistically significant.

**Conclusion:**

In the light of the previous findings, we concluded that numerical aberrations of chromosome 17 and p53 gene mutations as well as expression of p53 protein have enormous influence on various cellular processes including differentiation and carcinogenesis. Such knowledge provides an easy and simplified approach to prognosis predilection for OSCC.

**Virtual slides:**

The virtual slide(s) for this article can be found here: http://www.diagnosticpathology.diagnomx.eu/vs/13000_2015_232.

## Background

The development of oral squamous cell carcinoma (OSCC) depends on both environmental and genetic factors. Most oral cancer cases have had prolonged exposure to tobacco and alcohol, but these carcinogens cannot fully account for the development of cancers in these individuals [1]. Numerous studies have shown that tobacco causes damage of the cell DNA and alcohol reduces the effectiveness of DNA repairing that would be needed [2]. The accumulations of genetic abnormalities in carcinogenesis are divided into four phases: initiation, promotion, conversion and progression [3]. Cytogenetic analysis has detected an accumulation of genetic lesions in oral cancers [4]. Numerical changes in chromosomes 7 and 17 might be associated with an up-regulation of p53 gene, and could contribute to critical events in laryngeal carcinogenesis [4]. Moreover, human papillomavirus-associated oropharyngeal carcinoma (HPV-associated oropharyngeal carcinoma) was also reported recently [5]. Deactivation and unregulated expression of oncogenes and tumor suppressor genes might be involved in the pathogenesis of OSCC [6]. Molec-specific DNA probes, facilitate the confirmation of presumed chromosomal aberrations with high sensitivity and specificity. The acquisition of genetic instability is an essential step during carcinogenesis [7]. In most tumors, including OSCC, such a genomic changes result in alteration of chromosomal number and structure. A high frequency of chromosome 17 abnormalities has been reported in some human cancers such as breast carcinoma, colon carcinoma and bladder carcinoma [8-10]. Different studies revealed that cells with polysomy of chromosome 17 are significantly increased in squamous cell carcinoma the previous finding might indicate that chromosome 17 abnormalities seems to be correlated with carcinogenesis of OSCC [11].

The development and progression of human tumors often involves inactivation of tumor suppressor gene function [12]. The P53 gene, located on the short arm of chromosome 17p13, consists of 11 exons coding for a nuclear phosphorprotein, which can bind to specific DNA sequences acting as a transcription factor. Dysfunction in the P53 tumor suppressor gene is involved in the etiopathogenicity of OSCC [4]. The exact role of the p53 genetic alterations in different stages of the tumorigenic process is not completely established. The p53 gene has the capacity to induce repair of the damaged DNA by activating repair proteins and by stopping the cell cycle at the G/S regulation point, arresting growth of the cells. Another anti-cancer role of P53 is initiating apoptosis of a cell with irreparable DNA damage [13].

The aim of this study was to identify numerical aberrations of chromosome 17, deletion or amplification of P53 gene and to reveal possible correlations between these abnormalities and histological grading in patients with OSCC to be used as an easy and simplified prognostic marker.

## Methods

This study were performed retrospectively on forty anonymous paraffin embedded blocks diagnosed with a primary OSCC and clinical data were obtained without any personal information’s on those cases from the archives of faculty of Dentistry, Oral Pathology Department and the Oncology center of Mansoura University, Egypt between the years 2010 and 2013. Tumor samples were processed by usual techniques for inclusion in paraffin in the pathology lab of faculty of Medicine, Mansoura University. 3 micrometer-thick sections from the paraffin blocks were stained with hematoxylin-eosin for the establishment of the histopathological type and differentiation stage, based on the WHO International Classification of Diseases for Oncology (1990). Clinical staging was performed using the TNM Staging. Additional sections were prepared for immunohistochemical analysis and FISH technique evaluation. Five normal control slides obtained from mucosal specimens of non-oral cancer patients for this comparative study. Details of the cases are given in Table 1.

**Table 1:** **Clinicopathological variables of studied cases**

**Patients characteristics**		**Number**	**(%)**
**Age**	≤40 years	8	20%
	>40 years	32	80%
**Sex**	Male	28	70%
	Female	12	30%
**Site**	Tongue	16	40%
	Lip	4	10%
	Palate	12	30%
	Cheek	8	20%
**Clinical staging (TNM grading system)**	Stage I	4	10%
	Stage II	8	20%
	Stage III	16	40%
	Stage IV	12	30%
**Histological grade**	Well differentiated	20	50%
	Keratinizing moderately differentiated	8	20%
	Non-keratinizing moderately differentiated	4	10%
	Poorly differentiated	8	20%

### p53 immunohistochemistry

The sections were deparaffinized in xylene and rehydrated in alcohol. Endogenous peroxidase activity was quenched by immersion in a 1:4 solution of 3% hydrogen peroxide in methanol for 20 min, followed by rinsing several times in Tris-buffered saline (TBS). Slides were placed in Copin jars containing a 1:10 solution of target retrieval solution (Target Retrieval Solution 10×, pH9.0, Dako Cytomation S2367, Glostrup, Denmark) in distilled water and heated in a water bath at 98°C for 20 min to unmask the antigen. After the heating steps, the jars were allowed to cool for 30 min. Nonspecific binding was blocked by incubation with 5% bovine serum albumin (BSA) in TBS for 30 min at room temperature, the slides were incubated with the primary anti-P53 at a dilution of 1:200 in antibody diluent (Antibody diluent, Dako Cytomation) overnight at room temperature in a moist chamber. The slides were rinsed in TBS (3 times for 10 min each) and then incubated with secondary antibody (EnVision detection Kit Peroxidase/DAB Rabbit/mouse, Dako Cytomation) according to the manufacturer’s instructions. The slides were rinsed in TBS (3 times for 10 min each) and then stained with 3,3′-diaminobenzidinetetrahydrochloride (EnVision detection Kit Peroxidase/DAB Rabbit/mouse, Dako Cytomation). Sections were counterstained with Mayer’s haematoxylin solution. Negative controls were run in parallel, replacing the primary antibody with antibody diluent. The immunoreactive protein was localized in cancer cell nuclei.

### Digital image analysis

Slides were photographed using Olympus® digital camera installed on Olympus® microscope with 1/2 X photo adaptor, using 40 X objective. The images were analyzed on Intel® Core I3® based computer using VideoTest Morphology® software (Russia) with a specific built-in automated object counting routine for immunohistostain analysis. The immunoreactivity in the malignant cells in each section was graded according to the number of positively staining nuclei: ≤ 1% nuclei with a positive reaction are considered as a negative, > 1 ≤ 10% as low (+), >10% ≤ 50% as intermediate (++) and > 50% as high (+++).

### Interphase FISH analysis

Interphase FISH technique was done on thin formalin- fixed paraffin- embedded material. Using the Olympus BX 61, fluorescent microscope equipped with appropriate filters suitable for Fluorescein, Rhodamine and DAPI which present in Oncology Center, Faculty of Medicine, Mansoura University. Representative images were captured via a monochrome digital camera using Cytovision Image Capture software (Applied Imaging).

#### Technique

Interphase FISH technique was performed on tissue sections after optimization of the protocol using commercially available probe from Vysis, locus specific identifier LSA TP53/CEP 17 FISH Probe Kit which is intended to detect the copy number of the LSI TP53 probe Spectrum Orange target located at chromosome 17p13.1 and of the CEP 17 (17p11.1-q11.1 Alpha Satellite) probe Spectrum Green Dual Colour target located at the centromere of chromosome 17 according manufacturer’s protocol Abbott/Vysis (Vysis, Downers Grove, IL, USA). Sections were deparaffinized in xylene (3 times each for 5 min) and dehydrated in ethanol (3 times each for 3 min). Sections were pre-treated by placing the slides in 1xHCL (hydrochloric acid) 0.1 M for 20 minutes to avoid tissue autofluorescence, then the slides were placed in 2X SSC (Standard Saline Citrate) wash in a coplin jar for 3 minutes. The slides were then heated in microwave at 99°C in 1 mM sodium citrate buffer (pH 6.0) for 60 min, followed by 3 min in distilled water, 20 min in pepsin solution (Sigma P8038) (0.05 mg/mL pepsin solution in N HCL 0.01 M) at 37°C followed by washing in distilled water for 3 min and dehydrating for 1 min in increasing concentrations of alcohol (70, 85 and 100%) after which they were air-dried. Then 20 ul of 50% formamide hybridization buffer was added to the marked area on the slide and incubated at 90°C for 20 min on the HyBrite™ machine (Olympus Life Science). After that five μl of probe solution were applied to each slide, overlaid with a coverslip, which was sealed with rubber cement. Slides were denatured for 5 min at 73°C and hybridized for at least 16 h at 37°C in a Vysis HyBrite™ machine. Following hybridization, the slides were washed with 2 × SSC/0.1% NP40 at room temperature for 5 min then at 73°C with 0.4 × SSC/0.3% NP40 for 2 min and again at room temperature with 2 × SSC/0.1% NP40 for 1 min. After air-drying in the dark, slides were counterstained with 10 μl of 4′6′-diamino-2-phenylindole (DAPI II). A cover slip was applied and sealed with nail varnish. The slides were analyzed Using the Olympus BX 61, fluorescent microscope (Olympus Life Science) equipped with appropriate filters suitable for Fluorescein, Rhodamine and DAPI. Representative images were captured via a monochrome digital camera using Cytovision Image Capture software (Applied Imaging).

#### Interpretation of FISH results

The LSI P53 probe hybridizes to chromosome 17. The approximately 172 kb Spectrum Orange P53 probe contains the complete TP53 gene and is located at chromosome 17p13.1. The Spectrum Green CEP 17 probe is a control probe which hybridizes to the centromere region of chromosome 17p11.1-q11.1. All the patients’ subjects presented the numerical alterations in the chromosome 17 and the P53 gene. On hundred nuclei were scored under x100 magnification, using an oil immersion objective and the fluorescent microscope for each defined histological area from the tumor and tumor-adjacent epithelia, each nucleus being assessed for the chromosome copy number. The numerical aberrations of chromosome 17 varied from individual to individual. Specifically, only distinct isolated nuclei were counted. We interpreted as monosomy 17 if the mean number of green signals in analyzed cells for each subject was lower than two. Chromosome polysomy was defined as the fraction of the cells demonstrating three or more green signals in each nucleus. In normal cell, therefore there should be 2O2G signals. We interpreted as P53 gene deletion if the there is one orange signal in analyzed cells for each subject. Multiplication in P53 gene was defined as demonstrating more than 2 orange signals in each nucleus. In general according the control, cut off 5% was used.

#### Statistical analysis

Statistical analysis of the data was done by using Statistical Package for Social Science (SPSS) version 20.0 Qualitative variables were presented as number and percent. Quantitative variable were presented as mean ± SD. Kendall’s tau-b correlation was used to assess relations between variables. Significance was considered when P value < 0.05.

## Results

This study included forty cases of OSCC with a high tendency for occurrence in old male patients (all of the males were smokers). Most of the cases were outdoor workers (farmers & street venders). All the tumors were intraoral and the tongue was the most common site (40%). In relation to the TNM staging system, majority of cases were stage III and stage IV respectively (40%, 30%). Among the histological grades, well differentiated type was the most frequent (50% of all studied cases) (Table 1).

**Figure 1:**
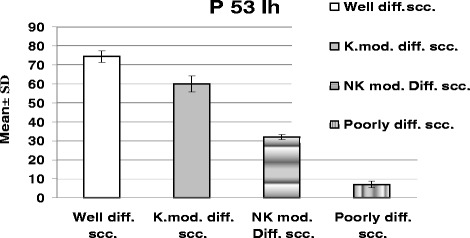
**Shows the mean ± SD of P53 immunohistochemical expression in different histological grades of the studied SCC cases.**

**Figure 2:**
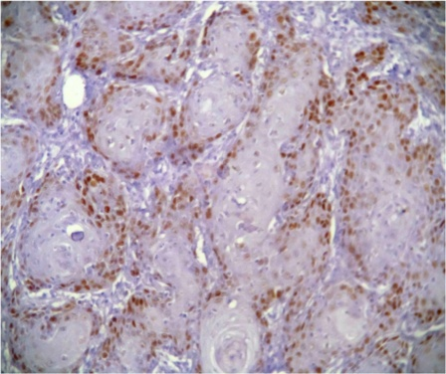
**Well differentiated SCC revealed intense immunoreactivity for p53.**

**Figure 3:**
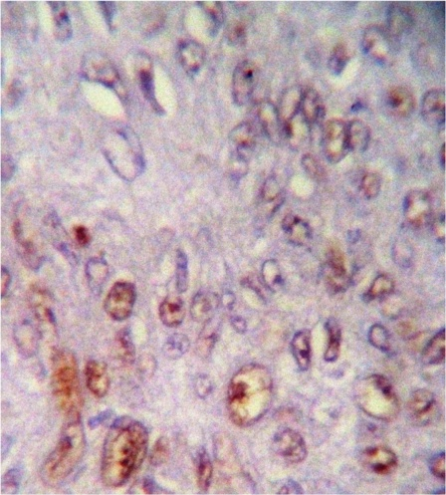
**Poorly differentiated SCC showed mild p53 immunoreactivity.**

**Figure 4:**
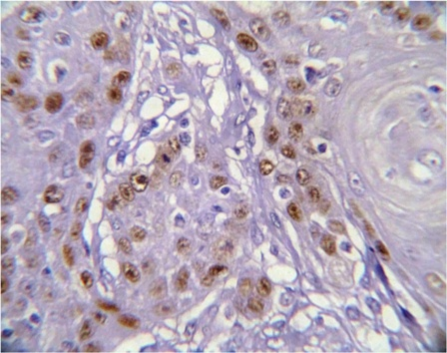
**Keratinizing moderately differentiated SCC revealed a moderate p53 immunopositivity.**

**Figure 5:**
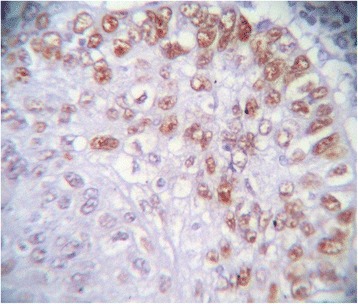
**Non- keratinizing moderately differentiated SCC showed moderate p53 immunoreactivity.**

Immunohistochemically, all studied cases showed a positive nuclear staining for the p53 protein with different indices (Figure 1). Well differentiated form of SCC showed the highest extent of P53 immunoreactivity (mean ± standard deviation (SD) = 74.40 ± 3.22) (Figure 2), whereas poorly differentiated cases showed lowest immunopositivity for p53 (mean ± SD = 7.00 ± 1.69) (Figure 3). In moderately differentiated SCC cases, the tumor staining for p53 was intermediate but with different extents in keratinizing and none keratinizing subtypes. The keratinizing moderately differentiated variant revealed a higher level of p53 immunoreactivity (mean ± SD = 60.00 ± 4.07) (Figure 4) than the non- keratinizing subtype (mean ± SD = 32.00 ± 1.15)(Figure 5). Statistical analysis of these findings showed a significant difference between all histological types of OSCC in relation to the immunohistochemical expression of p53 protein (Table 2).

**Table 2:** **Shows the mean ± SD of P53 immunohistochemical expression in different histological grades of the studied SCC cases (ANOVA)**

**Comparison**	**Well diff. SCC**	**Keratinizing moderately diff. SCC**	**Non-keratinizing moderately diff. SCC**	**Poorly diff. SCC**	**ANOVA P value**
**Mean ± SD**	74.40 ± 3.22	60.00 ± 4.07	32.00 ± 1.15	7.00 ± 1.69	0.000
**P1**		0.000	0.000	0.000	
**P2**			0.000	0.000	
**P3**				0.000	

All results are expressed as mean ± SD.

Non significant: at P >0.05.

Significant: at P < 0.05.

P1 = significance between Well differentiated SCC group and other groups.

P2 = significance between Keratinizing moderately differentiated SCC and other groups.

P3 = significance between Non Keratinizing moderately differentiated SCC and Poorly differentiated SCC.

**Figure 6:**
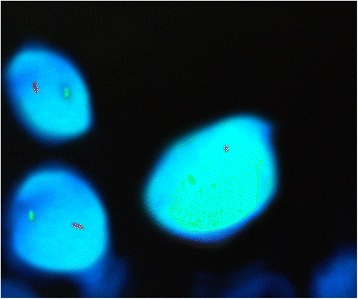
**FISH analysis using LSI P53 Spectrum Orange/CEP 17 Spectrum Green Probe.** One signal for each fluorochrome showing monosomy 17 and p53 deletion.

**Figure 7:**
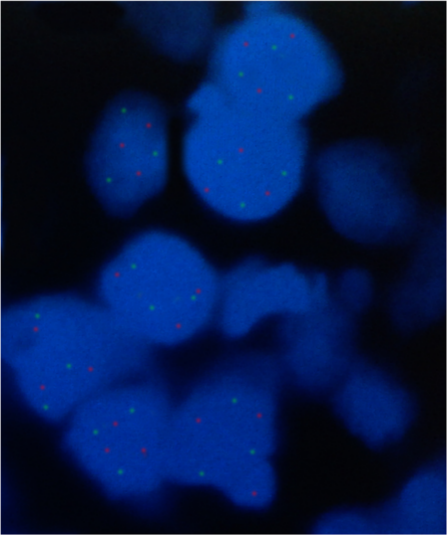
**FISH analysis using Vysis LSI TP53 Spectrum Orange/CEP 17 Spectrum Green Probe.** Three specific green signals fo trisomy 17 and three specific red signals for p53 amplification.

**Figure 8:**
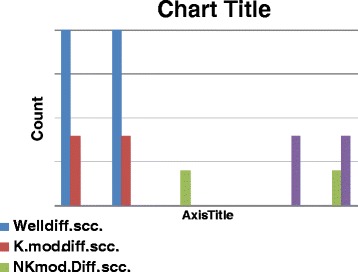
**Shows chromosome 17 abnormalities, P53 gene mutations in different histological types of the studied SCC cases.**

In the present study as shown in Tables 3 and 4; 28 subjects (70%) with chromosome 17 trisomy and only 8 subjects (20%) with chromosome 17 monosomy (Figures 6 and 7) were detected. Cases with chromosome 17 trisomy presented p53 gene amplification or multiplication while the cases with chromosome 17 monosomy revealed p53 gene deletion (Figures 6 and 7). All well differentiated SCC cases as well as the keratinizing moderately differentiated SCC type showed chromosome 17 and p53 gene amplification or duplication. On the opposite side, all poorly differentiated SCC cases exhibited chromosome 17 and p53 gene deletion (Figure 6). The non-keratinizing moderately differentiated variant presented a striking finding. They revealed p53 gene deletion while the chromosome 17 was normal (Figures 6 and 8).

**Table 3:** **The mean number of signals for chromosome 17 abnormalities and p53 gene mutations in different histological grades of the studied SCC cases**

**Grade**	**Number (total = 20)**	**Mean number of signals**	
		**Chromosome 17 abnormalities**	**P53 gene mutation**
**Well differentiated SCC**	20	3	3
**Keratinizing moderately differentiated SCC**	8	3	3
**Non-keratinizing moderately differentiated SCC**	4	2	1
**Poorly differentiated SCC**	8	1	1

**Table 4:** **Shows chromosome 17 abnormalities, P53 gene mutations in different histological types of the studied SCC cases**

**Comparison**		**Well differentiated SCC (No & %)**	**Keratinizing moderately differentiated SCC (No & %)**	**Non keratinizing moderately differentiated SCC (No & %)**	**Poorly differentiated SCC (No & %)**
**Multiplication**	Chromosome 17	20 (100%)	8 (100%)	0	0
	P53 gene	20 (100%)	8 (100%)	0	0
**Normal**	Chromosome 17	0	0	4 (100%)	0
	P53 gene	0	0	0	0
**Deletion**	Chromosome 17		0	0	8 (100%)
	P53 gene	0	0	4 (100%)	8 (100%)
Total number		20	8	4	8

The Spearman correlation test revealed a statistical significant positive correlation between chromosome 17 abnormalities and p53 gene mutations as well as with the immunohistochemical expression of p53 proteins (P = .000). Moreover, the positive association between p53 gene mutations and the expression of p53 protein was statistically significant (P = .000) (Table 5).

**Table 5:** **Shows the Spearman correlations between chromosome 17 abnormalities, P53 gene mutations and immunohistochemical expression of P53 protein in different histological types of the studied SCC cases**

**Comparison**		**Chromosome 17 abnormalities**	**P53 gene mutations**	**Immunohistochemical expression of P53 protein**
**chromosome 17 abnormalities**	r	1.000	.988	.794
	P value		.000	.000
**P53 gene mutations**	r	.988	1.000	.784
	P value	.000		.000

Significant : at P < 0.05.

## Discussion

Universally, it is accepted now that alterations in multiple oncogenes and tumor suppressor genes are the genetic basis for human carcinogenesis [12]. The p53 gene is the most frequent target of genetic alterations, being mutated in half of human cancers [13]. The p53 mutation usually shows clonality in cancer, therefore it has occurred in the early stage of carcinogenesis, as in OSCC [14]. A very recent research used low copy number of mitochondrial DNA (mtDNA) to predict worse prognosis in early-stage laryngeal cancer patients [15]. So far, prognosis prediction will be the focus of cancer researchers for the assurance of saving cancer patients lives.

In the present study, (40%) of the OSCC cases were found in the tongue. This is the same as compared to most of the other studies [16,17]. Moreover, it was found that (80%) of the cases were in the 40 year or above age group (n = 32). Overall male: female ratio of about 2.33: 1. This is in accordance with other studies, as reported by Idris et al. and Sugarman et al. [18,19].

According to Llewellyn et al. [20], SCC is not so frequent in young patients. Only 1% to 6% of SCC cases occur in patients under the age of forty indicating that the occurrence in children and adolescent was extremely rare [20,21]. Surprisingly, in our study, patients under 40 years of age were reported as 20% (n = 8). This shows that the incidence of oral cancer among young patients has markedly increased in our country. From our results most of the patients were exposed to sun light and male patients were smokers the previous information’s are in accordance with the carcinogenic effect of these two factors [20]. Finally, these results may be also due to the increase of nutritional deficiencies, pesticides pollution and drugs addictions in youngster.

Regarding TNM staging of SCC, most of patients presented in stage III (40%) and stage IV (30%), respectively. This is almost the same as described by HAQ M et al. [17]. This indicates that SCC in our country is detected or diagnosed mostly in advanced stages which were in contrast to Manuel et al. and Okada et al. who showed presentation of the cases at their early stages [22,23]. This might be due to the fact of high illiteracy rate, ignorance about the disease and poor referral system in our country. In addition, Shah et al., 2010 reported that oral cancers are diagnosed late (Stage III and IV) in Pakistan and need immediate public and professional attention [24].

Well differentiated was the most common histopathological type of OSCC in our study. This finding is in contrast to Haq M et al., whom found that poorly differentiated SCC was the most prevalent histological variant [17]. In accordance with the findings of our study, Valerie et al. reported that poorly differentiated subtype of SCC occurs much less commonly than well differentiated variant [25,26].

In the present study, it was found that a positive nuclear staining for the p53 protein with different indices in all studied cases. This finding is in agreement with Bai L, Zhu W-G 2006, whom suggested that p53gene is known to play a key role in all types of human cancers [27]. This could be attributed to the significant involvement of p53 gene in oncogenesis as p53 gene plays a pivotal role in regulation of the cell cycle and induction of apoptosis. In addition, it were noticed that the characteristic feature of the p53 gene mutational map is the high frequency of missense point mutations. Unlike many other tumor suppressor genes, more than 80% of p53 gene mutations result in single amino-acid substitutions which lead to the synthesis of a stable full-length protein rather than deletions. These missense mutations lead to the synthesis of a protein that lacks specific DNA binding site and accumulates in the nucleus of tumor cells [28]. Furthermore, it was found that p53 gene to be overexpressed in 63% of oral carcinomas [3]. From the previous finding we can hypothesize that accumulation of the P53 protein in an inactive form might has a role in the development of OSCC. There is now strong evidence that mutation not only abrogates p53 tumor-suppressive functions, but in some instances can also endow the mutant proteins with novel activities. Such neomorphic p53 proteins are capable of dramatically altering tumor cell behavior, primarily through their interactions with other cellular proteins and regulation of cancer cell transcriptional programs [29].

An interesting observation was noted in the present study that highly differentiated tumors had a high p53 immunostaining whilst poorly differentiated showed a significantly weaker immunohistochemical expression of p53 compared with well differentiated form. These results are consistent with other studies which have demonstrated that; statistically significant co-relation was seen between the histological grade and p53 gene overexpression. Also, histological parameters which indicated high cellular turnover were also significantly associated with p53 gene overexpression [30]. This might be interpreted as most of the mutations in the poorly OSSC is truncating mutations (deletions) which may lead to less P53 protein production and absence of its reactivity in the nucleus which in turn indicating the aggressive nature of the poorly differentiated tumo [31-33].

In our study, a statistical significant positive correlation was found between chromosome 17 abnormalities and p53 gene mutations. Cases with chromosome 17 trisomy presented p53 gene amplification or multiplication while the cases with chromosome 17 monosomy revealed p53 gene deletion. Furthermore, keratinizing SCC cases exhibited amplification of chromosome 17 and p53 gene while poorly differentiated exhibited chromosome 17 and p53 gene deletions. Interestingly, mutations in the p53 gene occur at different phases of the multistep process of malignant transformation [34]. In accordance, Kozomara et al. mentioned that p53 deletions are associated with a higher risk of relapse and contribute to an even worse prognosis of patients with OSCC [35]. Moreover, numerical changes in chromosomes 17 might be associated with an up-regulation of p53 genes, and could contribute to critical events in laryngeal carcinogenesis [4]. Other data from literature revealed that the frequency of cells with polysomy increased with histological progression suggesting that the biological factors might influence the rate of accumulation of genetic hits. Genomic instability may also lead to chromosome non-disjunction and to the generation of cells with zero, one, two, and three or more chromosome copies [36]. Also, altered p53 gene expression in premalignant lesions is associated with increased chromosomal polysomy [3]. Hence, the presence of cells exhibiting three or more chromosome copies (chromosome polysomy) might be considered a quantitative marker of ongoing or accumulated genomic instability in tumors [37,38].

This study revealed a statistical significant positive correlation between chromosome 17 abnormalities and the immunohistochemical expression of p53 proteins. Other previously reported findings suggest that a statistically significant correlation existed between p53 protein expression and polysomy 17 [36,39,40]. Furthermore, p53 protein overexpression was associated with the frequency of p53 mutations in tumor tissue, while p53 mutations, particularly missense and nonsense mutations might have some impact on survival rate [39]. This could be explained by Fritsche et al. who indicated that wild type p53 activation involves an increase in overall p53 protein level as well as qualitative changes in the protein through extensive post-translational modification, thus resulting in activation of p53-targeted genes [41]. Moreover, the immunohistochemically detectable high p53 gene expression levels reflect the increased half-life of the protein encoded by a mutated gene [42,43]. Also, Busby-Earle et al. concluded that somatic mutation in the hotspot regions of the p53 gene occurs infrequently in cervical carcinomas; that immunocytochemically detectable levels of p53 are also infrequent [44]. Upon these results, it is assumed that polysomy in chromosome 17 correlates with the mutation of p53 gene which results in an accumulation of aberrant p53 protein.

## Conclusion

In the light of the current study findings, we found that numerical aberrations of chromosome 17, p53 gene mutations as well as expression of p53 protein have massive influence on various cellular processes including differentiation and carcinogenesis as mutant p53 can induce an increased epigenetic instability of tumor cells, facilitating and accelerating the evolution of the tumor. From our results, we conclude that bad prognosis (aggressive tumors) is directly correlated with chromosome 17 aberrations and P53 deletion mutation. Finally, such knowledge provides an easy and simplified approach to prognosis predilection for OSCC.
